# Obesity, Diet, and Activity in relation to Asthma and Wheeze among Rural Dwelling Children and Adolescents

**DOI:** 10.1155/2013/315096

**Published:** 2013-09-26

**Authors:** Joshua A. Lawson, Donna C. Rennie, James A. Dosman, Allison L. Cammer, Ambikaipakan Senthilselvan

**Affiliations:** ^1^Canadian Centre for Health and Safety in Agriculture, University of Saskatchewan, 3641-103 Hospital Drive, Royal University Hospital, Saskatoon, SK, Canada S7N 0W8; ^2^Department of Medicine, University of Saskatchewan, Saskatoon, SK, Canada S7J 5B6; ^3^Canadian Centre for Health and Safety in Agriculture, University of Saskatchewan, Saskatoon, SK, Canada S7N 0W8; ^4^College of Nursing, University of Saskatchewan, Saskatoon, SK, Canada S7N 5E5; ^5^School of Public Health, University of Alberta, Edmonton, AB, Canada T6G 1C9

## Abstract

*Aims and Objectives*. We investigated associations between weight status, activity level, and diet with asthma or wheeze as well as the interrelationship between these factors. *Methods*. We conducted a case-control study of 6–18-year olds from 2005 to 2007. Cases (*n* = 87) were subjects reporting episodes or breathing medication use along with doctor-diagnosed asthma or wheeze in the past 12 months. Controls were randomly selected (*n* = 208) and without asthma or wheeze. Data regarding health outcomes, diet, and activity were obtained from questionnaire. Objectively measured height and weight were collected. *Results*. In the adjusted analysis, there was a trend (*P* = 0.07) towards an increased risk of asthma or wheeze associated with high fast food and/or pop consumption. Among cases, a significantly lower proportion (66%) classified as overweight participated in hard exercise in ≥9 of the past 14 days compared to those who were not overweight (86%). This pattern was not seen among controls (76% participating in hard exercise versus 78%, resp.). However, based on perceived weight status by the parent, the patterns were similar regardless of case-control status. *Conclusions*. Overweight status may negatively impact activity level among those with asthma or wheeze. Efforts should be made to encourage healthy food choices, and activity programming must consider the needs of overweight children with asthma.

## 1. Introduction

Asthma is a chronic inflammatory disorder of the airways that can be characterized by symptoms such as episodes of wheezing, breathlessness, chest tightness, and coughing with wheeze being the most common physical finding [1]. It is most prevalent among children [2, 3]. Childhood asthma results in a large amount of health care utilization and associated resource use leading to a huge impact on society [2, 4]. At an individual level, persons with asthma demonstrate lower quality of life compared to those without asthma [5, 6]. Part of this results from reduced participation and activity limitation, including school or work absenteeism. Among children, geographic trends in asthma prevalence have been reported with higher prevalence of asthma observed in westernized nations [7, 8] and in urban [9] and nonfarming [10] locations. In addition, there is evidence of temporal trends with increases in asthma prevalence occurring over the past few decades [3, 11].

The reason for geographic and temporal variation is unclear. The environmental factors have been considered as possible explanations for these trends, but a less examined and potentially important explanatory factor includes health behaviours and obesity. Associations with obesity have been the most studied of these alternate explanations. As with asthma, rates of obesity have been rising in recent years [12]. There is also evidence that being overweight or obese is associated with an increased risk of asthma or wheeze [9, 13, 14].

Various components of a child's diet have been associated with asthma or wheeze. Some foods, such as fish products [15] or vegetable and milk consumption [9], have been suggested to be protective of asthma, while other foods, such as hamburger consumption or “fast foods,” have been suggested to increase the risk of asthma [16]. Finally, there is conflicting evidence on whether physical activity is associated with asthma [17–19].

Despite these findings, few studies have examined the interrelationship between diet, physical activity, and weight status with regard to children with asthma or wheeze. What is more, these investigations in relation to asthma and wheeze are rarely considered in rural populations despite there being differences in obesity and health behaviours between urban and rural dwellers [20]. As mentioned, results of several studies suggest that farming or rural exposure is protective of asthma and allergic disease [10, 21, 22] although this is not entirely consistent [23]. Within Canada, the protective effect of rural or farm dwelling has ranged from negligible [24] to more than a 50% reduction in risk [25] as well as an inverse dose-response association [9]. Thus, there are substantial gaps in knowledge regarding weight status, diet, and activity and their interrelationships in rural areas, where health care access is lower, and there are different exposure patterns compared to urban areas. 

In this study, some of these issues were investigated using the data collected from Humboldt, Saskatchewan, and the surrounding area, which has been the site of four previous cross-sectional studies on lung health. The primary objective was to examine weight status, diet, and activity level in relation to asthma or wheeze among a rural dwelling population of children and adolescents. The specific research questions considered in our study were (1) what is the profile of weight status, diet, and activity levels among children with and without asthma? (2) Are characteristics of a child or adolescent's weight status, diet, or activity levels associated with having active asthma or wheeze? (3) How do health behaviours and weight status interrelate among children and adolescents with and without asthma?

## 2. Methods

### 2.1. Study Design and Population

The methods used in this study have been described elsewhere [26, 27]. In brief, we conducted a case-control study of children and adolescents living in and around the rural community of Humboldt, Saskatchewan, Canada, during the fall and winter seasons from 2005 to 2007 with some extended data collection in the spring. Rural schools in the same school district as schools within the community boundary of Humboldt were approached. Subjects were recruited from a previously conducted population-based cross-sectional survey of respiratory health of 6 to 18-year old children and adolescents completed in 2004. Potential cases comprised all subjects reporting either (1) wheeze in the past 12 months or (2) report of doctor-diagnosed asthma and at least one of health care utilization for asthma in the past 12 months, asthma medication use in the past 12 months, or asthma episodes in the past 12 months. Thus, the cases considered here were current asthma or wheeze cases.

For each case selected, two potential controls were randomly selected from among children who were not considered cases. Once selected, an invitation letter was mailed, and potential participants were contacted by telephone a maximum of seven times. Following contact, case and control status was confirmed by a screening questionnaire that enquired about asthma diagnoses, recent asthma, or wheeze events including symptoms, episodes, and breathing medication use. 

Data collection for the current analysis was based on an interviewer-administered case-control questionnaire, data from the original cross-sectional questionnaire, and saliva samples to assess exposure to tobacco smoke. The Health Research Ethics Board—Panel A (University of Alberta) and the Biomedical Research Ethics Board (University of Saskatchewan) approved the study as did the local school boards. Prior to taking part, parents and children completed consent and assent forms, respectively. 

### 2.2. Questionnaires and Operational Definitions

The questionnaires were based on previously validated and standardized questionnaires [7, 28, 29] and those used in previous respiratory health studies in Saskatchewan [30–32]. Information was collected on respiratory health, sociodemographic factors, general health, family history, birth characteristics, lifestyle, housing characteristics, and environmental exposures. 

Weight status was considered by two methods. The first was actual weight status. This was based on objectively measured height and weight. Height was measured against a wall using a fixed tape measure with subjects standing in socks on a hard floor. Weight was measured using a calibrated spring scale with subjects in socks and dressed in normal indoor clothing. From these measures, body mass index (BMI) was calculated based on the equation (BMI = weight (kg)/height (m)^2^) [33]. Weight status was classified based on categorization of body mass index (BMI) using values described by Cole et al. [34] which base the cut-off value as the predicted adult equivalent of 25 for overweight.

The second method of classifying weight status was based on weight status perceived by the parent. This was considered by the question “Do you consider your child to be: Underweight/Just about right weight/Overweight?.” If a parent responded with overweight, the child's perceived weight status was “overweight.” This question was based on a question used in the United States National Health and Nutrition Examination Survey III [35].

Physical activity level was classified based on parental response to the question: “How many of the past 14 days has your child done at least 20 minutes of exercise hard enough to make him/her breathe heavily and make his/her heart beat fast? (hard exercise includes, e.g., playing basketball, jogging, or fast bicycling and includes time in physical education class): None/1 to 2 days/3 to 5 days/6–8 days/9 or more days.” This was based on the Modifiable Activity Questionnaire for Adolescents [36]. Children were categorized as <9 days versus ≥9 days (High activity level).

Classification of diet was based on questions used in the International Study of Allergy and Asthma in Childhood questionnaire for dietary assessment [37] as well as an additional question on soft drink consumption. Dietary questions were based on “In the past 12 months, how often, on average, did your child eat or drink the following… Never/Occasionally/Once or twice per week/3 or more times per week” with a number of food categories considered. Soft drink consumption was based on the question “On average, how many soft drinks or pops does this child drink in a week?” Regular consumption of a given food was considered to occur if the child ate that food at least once or twice per week on average. The foods considered were based on findings from previous studies [9, 15, 16] and included vegetables, fruits, milk, and fish (including seafood). High soft drink consumption was considered to occur if the child drank 2 or more soft drinks or pops per week on average (the 75th percentile). As an indicator of poor diet, if the child was considered to have either regular soft drink or fast food consumption, then they were classified in the “Regular fast food/soft drink consumption group.”

The classification methods for each of the following potential confounders have been used in previous studies by our group when examining pediatric asthma [9, 24, 26, 27]. Subjects were classified into age groups (≤12 years versus >12 years) to be comparable with other childhood asthma studies where age ranges were typically between 6 and 12 years, [31, 38]. Season of testing was defined by the date of the home visit and was recorded as spring (March, April, and May), fall (September, October, and November), and winter (December, January, and February) to account for the potential differences in endotoxin and allergy levels by season. 

### 2.3. Collection and Analysis of Saliva Samples to Quantify Cotinine Levels

Tobacco smoke exposure was determined by salivary cotinine levels. Subjects were asked to spit into a specimen container without the use of gum, Teflon, or other materials that would stimulate the flow of saliva. Up to 5 mL of saliva was collected. Analysis for cotinine was conducted using saliva cotinine microplate enzyme immunoassay kits (Cozart plc, UK). Levels of cotinine (ng/mL) were categorized as high and low post analysis based on the median cotinine level (1.24 ng/mL) due to a highly skewed distribution of results that could not be normalized after log transformation.

### 2.4. Statistical Analysis

Analysis was completed using SPSS version 20. Throughout the analysis we considered statistical significance based on an alpha level of <0.05. Initially we described weight status and health behaviours descriptively to develop a profile of these characteristics in a rural population. This was completed through examination of agreement between the perceived weight status and actual weight status using percent agreement, Kappa statistics, and descriptive comparisons of mean BMI. We also considered frequencies and proportions among cases and controls to describe the population and used chi-squared tests to make statistical comparisons between these two groups. 

Next we measured the association between case-control status and dietary characteristics, activity level, and weight status using logistic regression. A multiple logistic regression model was used to adjust for potential confounders. Variables were chosen to be included in the model based on clinical and biological importance from the literature, statistical significance, or the effect that the removal of that variable had on the beta coefficient of the other variables in the model. To avoid colinearity, we fitted several models but only included one variable of a certain type of diet or weight status while adjusting for the other variables. The strength of the association was quantified using the odds ratio (OR) and 95% confidence intervals (CI). Statistical significance was assessed by comparing the observed *P* value to the aforementioned alpha level.

 In our final set of analyses we considered the interrelationship between each of the following health behavior variables: diet, weight status (actual and perceived), and activity level first descriptively then using chi-squared tests for proportions. Following this, we tested for statistical significance by using multiple logistic regression to adjust for potential confounders. These final sets of inter-relationship analyses were stratified by case-control status. Models were fitted to avoid colinearity between diet variables and included one diet variable at a time (Adjusted models 1 to 4). Also, models were fitted with one of actual weight status based on BMI (adjusted models 1 and 2) or perceived weight status (adjusted models 3 and 4) independently.

## 3. Results

Among eligible cases and controls, 322 children or adolescents and their parents agreed to participate in the study (participation rate = 43.4%). Of these, 208 were controls and 87 were cases. The remaining 27 children were excluded due to either missing data (*n* = 12) or they were cases who had asthma that was no longer considered current (*n* = 15). When analyses considering perceived weight status were conducted there were an additional 22 fewer participants due to missing data in this specific variable. Characteristics of cases and controls are presented in Table 1.

A high proportion of cases and controls were overweight based on objective measurements with a much lower proportion of parents perceiving their children to be overweight (Table 1). Among cases, there was 72.8% agreement between actual weight status and perceived weight status but with 25.9% of parents perceiving their children to not be overweight when in fact they were considered overweight by objective measures. This was similar among controls where there was 72.4% agreement between actual weight status and perceived weight status, with 25.5% of parents perceiving their children to not be overweight when in fact they were measured as overweight. This resulted in Kappa statistics of 0.28 for cases and 0.19 for controls indicating a poor agreement. When cases were adjusted for age, mean BMI in the actual overweight group was 24.5 and 26.6 in the perceived overweight group. Among controls, mean BMI in the actual overweight group was 24.9 and 25.4 in the perceived overweight group after adjusting for age.

There were no statistically significant differences between cases and controls in weight status, perceived overweight status, activity level, or dietary intake (Table 1) although there was a trend towards statistical significance with cases reporting a higher proportion with regular fast food/soft drink consumption than controls (65.5% and 54.3%, resp.; *P* = 0.08). Given the high proportion of having a regular diet of fruit, vegetable, and milk consumption, these variables were not considered in subsequent analyses. After adjusting for potential confounders, there were no statistically significant associations (*P* > 0.05) between any of the health behaviours or overweight status with case status (Table 2). While the directions of the associations were as expected, they tended to be weak based on the OR, with the exception of fast food and/or soft drink consumption. This was after fitting independent models to avoid potential co-linearity between weight status and dietary intake (models 1 to 4). Again, there was a trend towards an association for regular fast food/soft drink consumption (OR = 1.67, 95% CI = 0.95–2.95, *P* = 0.08).

**Figure 1:**
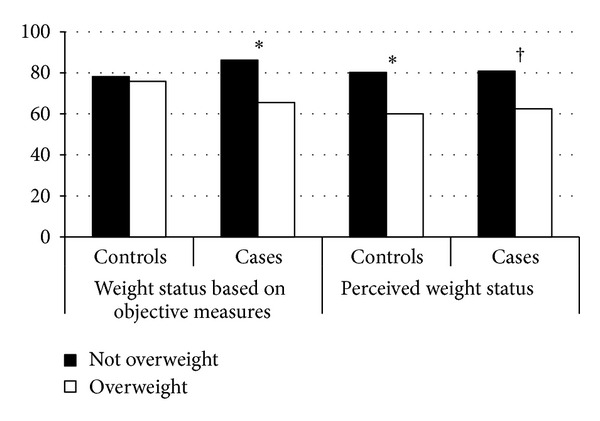
Proportion of children and adolescents with high activity levels who are defined as overweight based on body mass index and based on their parent's perception of overweight by case-control status. *P < 0.05 comparing overweight to not overweight after adjusting for sex, home air filter, maternal smoking during pregnancy, bare bedroom floor in the first year of life, age group, season of testing, tobacco smoke exposure, and high soft drink or fast food consumption. ^†^ *P* = 0.11 comparing overweight to not overweight after adjusting for sex, home air filter, maternal smoking during pregnancy, bare bedroom floor in the first year of life, age group, season of testing, tobacco smoke exposure, and high soft drink or fast food consumption.

When considering the interrelationships between the health behaviours and weight status including actual and perceived weight status, there was no statistically significant association between poor diet (regular fast food/soft drink consumption) and any of the other measures among cases or controls (Table 3). As seen in Figure 1, when considering physical activity levels, among cases, a higher proportion of children had high activity levels if they were classified not overweight compared to if they were classified overweight regardless of if the weight status was based on objective measures or perception, although this was not statistically significant when considering perceived weight status (*P* = 0.11). However, among controls, when using objective measures of weight status, there was not a statistically significant difference between those who were overweight and those who were not with regard to having high activity levels. There was a significantly lower proportion of controls with high activity levels when they were perceived to be overweight compared to when they were not perceived to be overweight (Figure 1).

## 4. Discussion

We sought to describe and examine weight status, diet, and activity levels in relation to asthma and wheeze among children and adolescents in a rural setting. We found that a large proportion of children and adolescents were overweight when considering objective measures of height and weight, yet a low proportion of parents perceived their child as being overweight. Health behaviours and weight status in this population did not show strong associations with asthma or wheeze, and children and adolescents with asthma who were also considered overweight were less likely to have a high activity level. 

 We found that close to one-third of children and adolescents in our study population were overweight or obese when using BMI to classify weight status. This is similar to levels found in previous studies looking at national samples of Canadian children and adolescents which report the prevalence of overweight and obesity to be between 18% and 39% depending on age, sex, and location of residence [12, 20]. This high prevalence of overweight classification should be addressed given the long-term negative health consequences of childhood obesity [39].

We also confirm previous studies that showed that parents generally underestimate the weight status of their children [35, 40]. Our results extended these previous findings to rural populations and found that parents of children with asthma or wheeze and those without asthma or wheeze do this similarly. In our study, approximately 80% of children who were overweight based on objective measurements were not perceived to be overweight by their parents. This discrepancy is higher than that observed in previous studies [35, 40]. Rural populations may be at increased risk for misclassifying children's weight status. Failure to recognize children as being overweight or obese could have serious health implications as action taken to address or prevent this condition and subsequent health effects cannot occur without first identifying that there is a problem.

 With regard to activity level and diet, it is more difficult to compare other studies given the large number of indicators and methods available. We found that the majority of the current study population engaged in high levels of physical activity. The level was higher than expected based on previous studies which found that approximately 50% to 57% of adolescents took part in high levels of activity [41]. However, regular physical activity levels in a separate study of Canadian adolescents were approximately 80%, which is similar to the levels we found in the current analysis [20]. Finally, it was difficult to assess dietary status as there appeared to be low sensitivity in the measures we used, possibly due to the small number of categories. Despite this, we were able to determine that over half of this population consumed fast food or high levels of soft drinks regularly. 

 We did not find strong associations between being overweight or obese, activity levels, or diet with asthma or wheeze although there is some indication of increased risk of asthma or wheeze associated with poor diet. Associations between obesity and asthma have been reported but often depend on personal characteristics such as sex and onset of puberty, with associations typically seen among females and those with earlier onset of puberty [13, 42]. The associations between physical activity with asthma and wheeze have been infrequently studied and with inconsistent results [17–19]. Results from the few diet studies have been inconsistent but have shown some foods to be protective [15], while other foods may increase the risk of asthma or wheeze [16]. The potential explanation for the increased risk of asthma or wheeze associated with poor diet in our study could be due to increased dietary sodium intake [43] and the increased systemic inflammation that results from eating items such as fast food regularly or in high quantities. It has been shown that higher levels of C-reactive protein are associated with a reduction in lung function [44], and a single high fat meal has been shown to significantly increase cholesterol and triglyceride levels as well as exhaled nitric oxide, a marker of airways inflammation [45]. We recently investigated the association between obesity and health behaviours with asthma in a national sample of adolescents in Canada [9]. In that study we found an increased risk of asthma associated with weight status and physical activity with a reduced risk of asthma associated with whole milk consumption and vegetable consumption. In the current study we did not find similar associations. Reasons for the differences may be that the earlier study focused only on adolescents, different outcome definitions were used because respiratory health was not the focus of the earlier study, and the earlier study included both urban and rural populations from across Canada. Despite these differences, most of the other associations were in the same direction between the two studies with several of them being similar in strength. Similarly, reasons for the differences in results between our current study and those of others could be the differences in exposure variables considered, study population, or study design. Given the large number of indicator variables available and differences in populations in these investigations, differences in results could occur through differential variability in exposures or outcomes or mechanisms of exposure-outcomes at different ages.

 We found that children and adolescents who have asthma or wheeze and who are considered overweight or obese were less likely to participate in high levels of activity. This was regardless of weight status based on objective measures or parent perception. In contrast, lower activity levels were only present among controls when overweight was defined by parent perception. Children and adolescents whose parents perceived them to be overweight had a higher mean BMI than those children who were classified as overweight based on objective measures. It could be that higher BMI could result in physiologic limitation leading to reduced activity among children. Children and adolescents with asthma may experience these changes at a lower level of BMI, resulting in differences between cases and controls. Although there is some inconsistency, children with asthma have been reported to have lower activity levels than children without asthma [46]. Among adults, increasing BMI was associated with reduced likelihood of exercise defined by a number of methods and consistently in a dose-response manner [47]. Studies to date have not considered the characteristics of overweight/obese and asthma status in a combined fashion among children. Our findings suggest that this is a population deserving special attention for activity programming. As it is, asthma is a barrier for exercise in children [46]. This especially true since being overweight/obese can worsen asthma outcomes. If asthma is less controlled or outcomes worsen, there is the possibility that children with asthma will be even less likely to participate in activity, potentially further increasing overweight/obese status. 

This study addresses notable gaps in knowledge in that few studies have investigated health behaviours and overweight/obesity in rural populations, especially in relation to asthma or wheeze despite indication that rural dwellers are at increased risk of being overweight or obese. In an earlier study we found the prevalence of overweight or obese in an adolescent Canadian population to be approximately 23% overall, and the prevalence of overweight or obese in rural areas was significantly higher (approximately 28%) than in large metro areas (approximately 19%) [20]. Given the high prevalence of being overweight or obese and the higher levels in the current study population who reside in rural areas, we highlight the need to recognize and address overweight and obesity issues in rural regions. Rural dwellers are a unique population with different exposures, social structure, socioeconomic structure, and access to health care, and specific focus should be made to promote healthy lifestyles in these regions.

The major limitation in this study is the lack of temporality. Due to the cross-sectional nature of the data, we are unable to determine if poor weight status resulted from lower activity levels or if lower activity levels resulted from poor weight status. Similarly, we are unable to comment on whether asthma or wheeze preceded the lower activity levels among those with a poorer weight status or if lower activity levels with poor weight status preceded asthma or wheeze. Regardless the temporal relationship, it is clear that children and adolescents who are overweight and who have asthma or wheeze represent a group of children with lower activity levels, and this should be considered a public health focus. Another limitation is that this was a secondary analysis based on data collected for a children's lung health study. Because of this, some variables of interest may not have been collected and alternate methods of assessing health behaviours and weight status were not used which may have resulted in misclassification. However, we did use objectively measured height and weight as well as standardized measures of perceived weight status and activity where the question we used for activity has been well validated. Our dietary measures may not have had as much sensitivity as desired but have been used in other assessments of diet and asthma. We did not have information on puberty status, another potential confounder. Data regarding this stage of life was not collected and unfortunately we were unable to control it. While we controlled age, we cannot rule out that there may be some results explained by confounding by puberty status.

 In conclusion, we report on several findings that warrant further examination and should be further addressed in the population. First, we found that there are high levels of being overweight in this rural population and parents often do not accurately perceive their children to be overweight or obese. Identifying children who are overweight or obese is the first step in addressing the condition, which can have long-term negative health consequences. Second, efforts should be made to encourage children to avoid unhealthy food choices. Finally, we found that overweight status may negatively impact activity levels and that this may be especially true among children with asthma or wheeze. As such, activity programming should consider the needs of overweight children with asthma specifically. Health behaviours and weight status of children in rural areas are an area in need of further research and practical application.

## Figures and Tables

**Table 1:** Characteristics of the cases and controls.

	Controls *n* = 208	Cases *n* = 87	*P* value
Personal characteristics			
Female (%)	60.6	35.6	<0.001
Parents with > high school (%)	39.4	37.9	0.81
With allergic disease (%)	33.2	73.6	<0.001
Family history of asthma (%)	7.2	23.0	<0.001
Maternal smoking during pregnancy (%)	9.1	20.7	0.006
Early respiratory illness (%)	8.7	18.4	0.02
High tobacco smoke exposure (%)	51.0	49.4	0.81
Age, years (mean, SD)	11.5 (3.2)	11.1 (2.9)	0.31
Weight status			
Actual overweight based on BMI (%)	29.8	33.3	0.55
Perceived overweight (%)	7.8	9.9	0.58
Activity levels			
Higher activity level (%)	77.4	79.3	0.72
Dietary intake			
Regular fast food and/or soft drink consumption (%)	54.3	65.5	0.08
Regular seafood consumption (%)	26.4	25.3	0.84
Regular vegetable consumption (%)	99.0	98.9	0.88
Regular fruit consumption (%)	99.5	98.9	0.52
Regular milk consumption (%)	95.2	96.6	0.60

**Table 2:** Prevalence of lifestyle characteristics and unadjusted and multiple logistic regression* results examining the associations between weight status and health behaviours with asthma or wheeze.

	Unadjusted OR (95% CI)	Adjusted models			
		Model 1 OR (95% CI)	Model 2 OR (95% CI)	Model 3 OR (95% CI)	Model 4 OR (95% CI)
Weight status					
Not overweight	1.00	1.00	1.00		
Overweight	1.17 (0.69–2.01)	1.15 (0.63–2.10)	1.15 (0.63–2.08)		
	*P* = 0.55	*P* = 0.65	*P* = 0.66		

Perceived weight status					
Not overweight	1.00			1.00	1.00
Overweight	1.29 (0.53–3.18)			1.63 (0.59–4.53)	1.59 (0.57–4.41)
	*P* = 0.58			*P* = 0.35	*P* = 0.38

Hard activity levels					
<9 days in two weeks	1.00	1.00	1.00	1.00	1.00
≥9 days in two weeks	1.12 (0.61–2.06)	1.37 (0.69–2.72)	1.38 (0.70–2.72)	1.42 (0.69–2.93)	1.43 (0.70–2.95)
	*P* = 0.72	*P* = 0.36	*P* = 0.36	*P* = 0.35	*P* = 0.33

Fast food and/or soft drink consumption					
Low	1.00	1.00		1.00	
Regular	1.60 (0.95–2.69)	1.67 (0.95–2.95)		1.55 (0.86–2.79)	
	*P* = 0.08	*P* = 0.08		*P* = 0.15	

Fish and seafood consumption					
Low	1.00		1.00		1.00
Regular	0.94 (0.53–1.67)		0.84 (0.45–1.58)		1.00 (0.52–1.92)
	*P* = 0.84		*P* = 0.60		*P* = 1.00

*All models are adjusted for sex, presence of a home air filter, maternal smoking during pregnancy, bare floor in the bedroom in the first year of life, age group, season of testing, tobacco smoke exposure, and each of the variables listed in the column.

**Table 3:** Interrelationship between fast food and soft drink consumption, weight status, and activity level by case-control status*.

	Controls		Cases	
	Low fast food and soft drink consumption (*n* = 95) %	High fast food and soft drink consumption (*n* = 113) %	Low fast food and soft drink consumption (*n* = 30) %	High fast food and soft drink consumption (*n* = 57) %
Hard activity level				
<9 days in two weeks	24.2	21.2	20.0	21.1
≥9 days in two weeks	75.8	78.8	80.0	78.9
Weight status based on BMI				
Not overweight	70.5	69.9	66.7	66.7
Overweight	29.5	30.1	33.3	33.3
Perceived weight status				
Not overweight	91.0	93.2	89.3	90.6
Overweight	9.0	6.8	10.7	9.4

*There were no statistically significant associations.
